# Social networks, work and network-based resources for the management of long-term conditions: a framework and study protocol for developing self-care support

**DOI:** 10.1186/1748-5908-6-56

**Published:** 2011-05-29

**Authors:** Anne Rogers, Ivaylo Vassilev, Caroline Sanders, Susan Kirk, Carolyn Chew-Graham, Anne Kennedy, Joanne Protheroe, Peter Bower, Christian Blickem, David Reeves, Dharmi Kapadia, Helen Brooks, Catherine Fullwood, Gerry Richardson

**Affiliations:** 1 Health Sciences Research Group, and Collaboration for Leadership in Applied Health Research and Care (CLAHRC) for Greater Manchester, School for Community Based Medicine, University of Manchester, Manchester, UK; 2 Health Sciences, University of York, YO10 5DD, UK

## Abstract

**Background:**

Increasing the effective targeting and promotion of self-care support for long-term conditions requires more of a focus on patient contexts and networks. The aim of this paper is to describe how within a programme of research and implementation, social networks are viewed as being centrally involved in the mobilisation and deployment of resources in the management of a chronic condition. This forms the basis of a novel approach to understanding, designing, and implementing new forms of self-management support.

**Methods:**

Drawing on evidence syntheses about social networks and capital and the role of information in self-management, we build on four conceptual approaches to inform the design of our research on the implementation of self-care support for people with long-term conditions. Our approach takes into consideration the form and content of social networks, notions of chronic illness work, normalisation process theory (NPT), and the whole systems informing self-management engagement (WISE) approach to self-care support.

**Discussion:**

The translation and implementation of a self-care agenda in contemporary health and social context needs to acknowledge and incorporate the resources and networks operating in patients' domestic and social environments and everyday lives. The latter compliments the focus on healthcare settings for developing and delivering self-care support by viewing communities and networks, as well as people suffering from long-term conditions, as a key means of support for managing long-term conditions. By focusing on patient work and social-network provision, our aim is to open up a second frontier in implementation research, to translate knowledge into better chronic illness management, and to shift the emphasis towards support that takes place outside formal health services.

## Introduction

The increase in the number of people living with long-term conditions (LTCs) and the high cost of providing services and support for long-term-condition management (LTCM) has highlighted the need for a greater focus on developing a variety of means of self-care support and behaviour change. As part of a wider agenda about public health and patient involvement [1], self-care support strategies have been identified as potentially benefiting 70% to 80% of people with LTCs.

At the level of policy, the benefits associated with self-management support include patient empowerment, increased self-efficacy, changes in behaviour, and a reduction in utilisation of healthcare resources. However, self-care strategies used in healthcare settings are in their infancy and currently operate with an equivocal evidence base regarding long-term change and are not always appropriately targeted, detailed or sufficiently person-centred to be constructive for patients [2]. The current dominant approach to self- management tends to focus on psychological mechanisms of behavioural change, may be excessively centred on individuals' capacity and responsibility to initiate and sustain strategies for self-management and often fail to take into account the social context of those living in disadvantaged circumstances [3]. The existing evidence suggests that, more attention needs to be placed on the contexts, resources, practices, priorities, and networks of patients living with a chronic condition in order to identify the nuanced ways in which self-care support and LTCM can be integrated into the open systems [4]^i ^of people's everyday lives^ii^.

The process of translation and implementation requires knowledge to be developed that addresses the policy challenge of improving the care for people with LTCs, revisiting the key questions that need to be asked and the ways in which the knowledge that is generated can be filtered into specific interventions. Applied health research, with a focus on translating research and implementation into practice [5], forms the basis of the Collaboration for Leadership in Applied Health Research and Care for Greater Manchester (CLAHRC).

The focus of the research agenda described here is on developing research aimed at constructing, adapting, and implementing strategies for self-care support for socially disadvantaged people with vascular conditions (diabetes, heart disease, kidney disease, and stroke). We are eliciting people's needs in order to develop support strategies that can be evaluated and added to an existing evidence-based approach to guided self- management support [6-8]. The programme incorporates an emphasis on considering responses to chronic-illness states as problems of action (of self and others) through investigating people's everyday life, identifying networks implicated in self-care, and on exploring the manner in which home and work impact the management of LTCs. Specifically, our programme aims to:

• identify the ranges of social economic and cultural resources that individuals draw on in responding to long-term health conditions;

• assess lay peoples' systems of support and access to resources that influence engagement with services, information, and coping strategies;

• identify how community social-capital resources (*e.g.*, economic, cultural, political, symbolic) are used (in positive and negative ways) for pursuing personal and collective goals and linked to LTCM;

• consider the ways in which networks and lay knowledge contribute to and inform the design of effective self-care interventions.

## Conceptual framework

Recent developments in self-management support emphasise understanding and improving individual's knowledge and capabilities and interactions with healthcare systems^iii^. This focus requires a complimentary understanding of the capabilities, resources, and health-related practices as an integral part of peoples' social networks^iv ^which are impacted upon by wider determinants of health. For example, class-related cultural resources interact with economic and social capital in the social structuring of people's health chances, choices, and the unequal distribution of health outcomes [7]. There is also a need to explore how professionally defined priorities of LTCM are translated, acted upon, and resourced outside of the consultation. In order to address these questions, we build on four conceptual approaches: (1) social networks, (2) illness work, (3) normalisation, and (4) the whole systems informing self-management engagement (WISE) approach. Framed in this way, questions related to changes in self-care practices and health behaviours bring into view ideas about implementing workable, personally sensitive strategies for self-management that recognise the use of available personal, community, and institutional resources that may more adequately address the needs of socially disadvantaged people.

### 1) Social networks and systems of support

**Figure 1:**
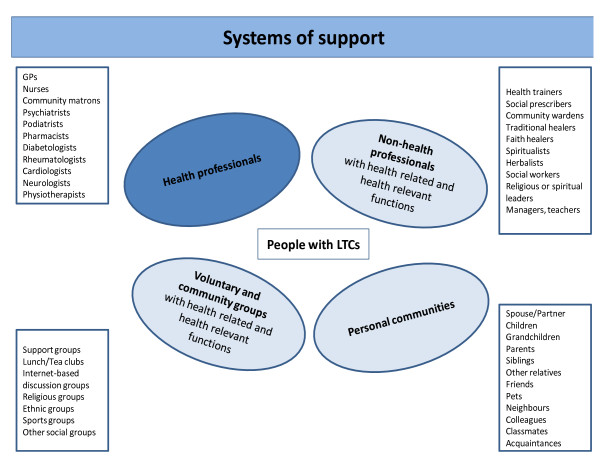
**Systems of Support for Long term conditions**.

We define social networks [9,10] as 'networks of networks' or 'systems of support' (see Figure 1). These have been operationalised and used empirically in studies of the family [11], ageing [12], and friendship [13] and could be fruitfully applied to LTCM. The advantages of using a broad definition of networks is that it offers a means to explore the functions and limitations of different types of support and affinities between types of illness-related work, relationships, and community belonging. Whilst traditionally case and disease management remain the province of health professionals, a social-network approach means that the main focus of self-management shifts to the person with the condition, members of their personal communities, support and community groups, nonhealth professionals, and, to a more limited extent, health professionals.

The distribution of the responsibilities for LTCM between groups of involved actors outside of the immediacy of the health service is not always made explicit and is more diffused if networks are taken into consideration. Once a broader set of relationships is taken into consideration, then a wider of view of what is relevant to self-care also emerges. The latter is likely to implicate a combination of the person with the condition, members of their personal communities, community groups, health professionals, and nonhealth professionals, as well as what can be negotiated from within the existing health system. The latter is further shaped by the social inequalities inherent in the health service and broader social system. If these factors, having been identified and empirically mapped, are taken into consideration, an alternative distribution of and nature of responsibilities for LTCM is likely to emerge. This could mean making changes to existing systems of support; devising new ways of engaging with self-care such as creating new roles for non-health professionals, extending services and support provided by voluntary and community groups, and acknowledging and/or extending the involvement of people with LTCs and members of their personal communities.^v^

**Figure 2:**
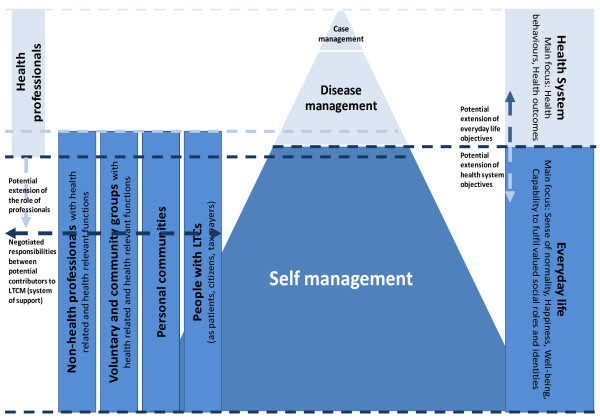
**Self Management, Chronic Illness and Social Networks**.

It also implicates a change in priorities. We know that within the context of the consultation, the concrete circumstances and priorities of individual patients may not always be adequately addressed by health professionals. A shift in focus to a broader network may mean that elements of professionally focused outcomes (*e.g.*, persuading patients to adopt desirable health behaviours) may at times be of secondary importance to patients in their everyday lives, compared to a sense of normality and well-being for themselves and for others (see Figure 2 patient-focused outcomes).

### 2) Illness management and types of work

The scope of the potential involvement of different providers of illness management requires a clearer conceptual understanding of the practices of illness management as they take place in everyday life. Here we draw on the sociology of chronic illness of Anselm Strauss [14,15] in order to distinguish *illness work *(which consists of crisis prevention and management, symptom management, and diagnostic-related work) from *everyday work *(housekeeping and repairing, occupational work, child rearing, sentimental work, eating) and *biographical work *(related to the reassessment of personal expectations, capabilities, and future plans) [15]. With the exception of biographical work, the other constructs, which are action oriented, are underidentified and tested empirically and are, thus, insufficiently specific about the contexts, resources, practices, and networks that fall within the ambit of 'work'. This results in 'work' becoming something of a procrustean bed that is not anchored in actual networks, resources, relationships, service use, healthcare, and social contexts, and trajectories of illness [16].

Our research programme builds on Corbin and Strauss's work by further distinguishing between five types of illness work: (1) contingency/improvisation: 'work that gets things back "on track"'; (2) translation, mediation work: the translation of abstract knowledge into practical knowledge and then into practice; (3) co-ordination work: negotiations regarding the ways in which work is done, who does what, when, how, and why; (4) advocacy work: the negotiation of contributions and the work done by others on one's behalf; and (5) emotional work: work related to comforting when worried or anxious.

### 3) Normalisation of illness-management practices

Illness management involves the adoption of a set of new practices, and changes to familiar ones. Changes that follow from a diagnosis of chronic illness are to a lesser or greater extent disruptive of familiar and comforting everyday routines, valued identities, and social roles. We draw on the normalisation process theory [17,18] to identify the key processes involved in adopting new and sustaining existing illness-management practices in everyday life. These include the formal and informal, visible and invisible work that is involved in the engagement, sense-making, and appraisal of illness-related practices; they are done both individually and collectively, and changes affect the individuals with the condition as well as members of their social networks. This framework is designed to help identify factors that promote and inhibit the implementation of new innovations in healthcare through identification of the factors influencing the introduction of these changes in contexts where negotiations are characterised by asymmetries of power and knowledge and to offer understandings of continuities and discontinuities in behaviours and processes involved in sustaining behaviour change [17]. Here our focus is on 'open systems'. This includes understanding the strength and interaction of 'asset' flows, especially social assets (*e.g.*, social capital), at critical points in the life course (*e.g.*, the onset or period of living with a chronic illness). More broadly, changes in existing practices and the introduction of new ones involves a multilevel negotiation between what is desirable and done collectively, who is doing what, and how are responsibilities shared. The ideological and normative frames within which material and discursive practice take place are difficult to separate and this raises questions about responsibility (who should do what, *e.g.*, politicians or experts) and in reconciling contradictory objectives within policy and practice, such as, for example, between professionally focused outcomes (*e.g.*, good biomedical indicators, appropriate health behaviours) and patient-focused outcomes (*e.g.*, sense of normality in everyday life, well-being) [19].

### 4) Multilevel approach to illness management

Drawing on and extending the WISE approach, we aim to identify the processes implicated in LTCM operating at different levels (patients in social context, professionals, and the system/organisation) in order to identify gaps in existing support for people with chronic conditions, which will inform new multilevel interventions. The WISE approach to self-care support combines lay and evidence-based knowledge with patient-centred consultations and flexible access arrangements to health services. It has been implemented and evaluated in National Health Services (NHS) contexts via a dedicated programme of research [18,19]. The main emphasis to date of the WISE approach has been on changes within the healthcare system, improving the communication and understanding in patient-professional interaction, and addressing the unequal power, knowledge, and competing priorities surrounding illness management (see Figure 2). Within this current programme of work, extend the WISE approach to develop a broader social/population view on LTCM.

## Stages of the research programme

The research programme is divided into three phases. These are centred on evidence synthesis and scoping the literature, empirical work, and developing and evaluating interventions.

## Scoping the literature

Prior to carrying out empirical work, we synthesised existing research in two reviews [20,21] (one on information-based interventions and the other on social networks). The evidence from our review of information-based interventions indicates that informational strategies that adopt a relativist, user-centred approach use lay language, and reflections of real-life experiences are more tailored to address socio-cultural contexts and are likely to have the most impact with people in socially disadvantaged groups. This suggests that developing interventions that include community-level strategies might produce favourable outcomes. Indeed, in our review of the social-networks literature, we found that social networks are widely implicated in LTCM through shaping and understanding normalcy and deviance, knowledge and narratives, the locus of individual responsibility, referrals, consultations, and how illness is managed by others. However, whilst the review indicated that social networks play an important part in the management of LTCs, we found that the notion of social networks has tended to be narrowly defined and is primarily used as a way of acknowledging the significance of context. There is insufficient discussion in the literature of the *types *of networks that support or undermine self-care and of network properties, as well as a lack of understanding of the processes involved that underpin the development of new interventions. This points to the need for new LTCM interventions to be delivered for translation and implementation through people's existing social networks, and it has also shaped the protocol developed for phase two of the research.

## Empirical study: a survey with nested qualitative study

The empirical study is being developed in six study areas in Greater Manchester. Three hundred patients with diabetes and heart disease will be recruited and selected as randomly sampled from general practice (GP) disease registers. A further purposeful sample of 30 participants with diabetes or heart disease will be recruited from groups who were underrepresented in the GP sample.

The questionnaire being used consists of two broad sections. Section one was a postal questionnaire and included questions on sociodemographic background, medical conditions and health status, use of self-care and self-care support, and a set of validated measures on aspects of social capital and social support.

A second survey instrument was administered and audio-recorded and included a set of questions on the patient's social network and the perceived support provided by carers, relatives, friends, neighbours, and statutory and voluntary services. The study aims to:

• understand and profile the networks and systems of support of people with LTCs;

• explore the relationship (contradiction, compatibility, and substitutability) between different types of resources within networks;

• examine how networks are related to different professional-focused and patient- focused outcomes.

## Development and evaluation of interventions

Drawing on the WISE approach and our empirical findings, we will develop interventions that aim to address LTCM as (a) a part of people's everyday life; (b) associated with the relationships between patients and health professionals; (c) related to the process of service funding, commissioning, and delivery; and (d) related to the links between different providers of health-relevant support (including professionals, voluntary, and community resources). Given the emphasis in our earlier studies on changes in the healthcare system, patient-professional interaction, and organisational culture [22,23], the interventions that we will be aiming to develop in this study will emphasise the functions and properties of systems of support for LTCM outside services *with *their links to the healthcare system. We will evaluate the acceptability, effectiveness, and economic efficiency of the interventions, while keeping a clear distinction between professionally defined and patient-defined priorities.

Further details describing the methods used in this research programme will be developed in separate publications.

## Discussion

The establishment of the nine CLAHRCs in England represents a shift in implementation research that focuses on how evidence can be translated into practice. This is taking place in the context of a publicly funded health service that is required to engage with national policy, as well as established professional ways of working. In this programme of research, we have recognised the need to increase the capacity of healthcare providers to apply evidence in expanding the ability of the NHS to promote LTCM. However, our approach also makes a distinction between what is provided from within *health services *and the need to focus on implementation and translation *outside *the NHS. In this respect we have chosen to focus on types of chronic illness and on the roles played by and resources of personal communities, local and community groups, health and non-health professionals, as well as people with LTCs. Within this, social networks are seen as a way of mapping a typology with which to gauge where and when the implementation of self-care support is likely to be most appropriately targeted. Focusing on social networks in this way offers an opportunity to assess what kinds of support people with LTCs value and is intended to recognise the important but often hidden roles played by people and groups within personal networks in supporting LTCM. These forms of support are sometimes less obviously health related according to traditional definitions but nevertheless give a sense of purpose, belonging, and well-being, which have significant knock-on effects for people with LTCs. Whether and what extent social networks might be used to implement self-care support in an efficient, effective, and acceptable way is a second objective to be achieved over the five years of the CLAHRCs. While previous research and implementation of patient-involvement strategies have been equivocal, by focusing in-depth on patient work and social-network provision, our aim is to open up a second frontier in implementation research, to translate knowledge into better chronic illness management, and to shift the emphasis towards healthcare that takes place outwith the confines of traditional health service delivery.

## Abbreviations

LTC: Long Term Condition Management

## Competing interests

AR is an Associate Editor of Implementation Science. All decisions on this manuscript were made by another senior editor. The author(s) declare that they have no other competing interests.

## Authors' contributions

All authors were involved in different stages of the study design.

All authors read and approved the final manuscript.

## End Notes

^i ^According to Bhaskar [4], closed systems reveal or disclose the functioning of mechanisms or powers independent of other intervening causes that tend to clothe or hide the powers of various entities. Open systems are messier; instead of processes being *isolated *from other events, powers operate without producing a particular effect.

^ii ^For example, there is evidence that community-level strategies improve the quality and availability of health-related resources and are important factors for the success of healthcare interventions [4].

^iii ^Our previous research show that the introduction of a guided self-management strategy within health service settings reduces consultations in primary care and secondary care, increases subjective well-being on the part of patients (*e.g.*, perceived reduction in symptoms), and increases people's ability to cope with their condition.

^iv ^One of the unanticipated benefits of self-management programmes such as the Expert Patients' Programme has been the potential of group activities to reduce social isolation through enabling contact and support from fellow course participants [5]. What appears as an unintended consequence of the Expert Patients' Programme nevertheless resonates with literature that suggests that crucial elements of self-care support lie outside the confines of both the individual and traditional health services [7,10]

^v ^None of these are mutually exclusive; for example, a redistribution of existing resources to impact on barriers for self-care and/or extending the role played by community groups and members of personal communities may be implicated alongside the reconfiguration of the roles that health professionals play in LTCM.
